# Comparative analysis of *Cucurbita pepo* metabolism throughout fruit development in acorn squash and oilseed pumpkin

**DOI:** 10.1038/hortres.2016.45

**Published:** 2016-09-21

**Authors:** Lindsay E Wyatt, Susan R Strickler, Lukas A Mueller, Michael Mazourek

**Affiliations:** 1 School of Integrative Plant Sciences, Section of Plant Breeding and Genetics, Cornell University, Ithaca, NY 14850, USA; 2 Boyce Thompson Institute for Plant Research, Ithaca, NY 14853, USA

## Abstract

Both the fruit mesocarp and the seeds of winter squash can be used for consumption, although the focus of breeding efforts varies by cultivar. Cultivars bred for fruit consumption are selected for fruit mesocarp quality traits such as carotenoid content, percent dry matter, and percent soluble solids, while these traits are essentially ignored in oilseed pumpkins. To compare fruit development in these two types of squash, we sequenced the fruit transcriptome of two cultivars bred for different purposes: an acorn squash, ‘Sweet REBA’, and an oilseed pumpkin, ‘Lady Godiva’. Putative metabolic pathways were developed for carotenoid, starch, and sucrose synthesis in winter squash fruit and squash homologs were identified for each of the structural genes in the pathways. Gene expression, especially of known rate-limiting and branch point genes, corresponded with metabolite accumulation both across development and between the two cultivars. Thus, developmental regulation of metabolite genes is an important factor in winter squash fruit quality.

## Introduction

*Cucurbita pepo* is the most genetically and morphologically diverse species within *Cucurbita*, spanning two subspecies, *C. pepo* ssp. *pepo* and *C. pepo* ssp. *texana*. Market classes of *C. pepo* include decorative gourds and pumpkins and oilseed pumpkins, the fruit of which is not consumed, summer squash and zucchini that are eaten at an immature stage, and acorn and delicata winter squash that are consumed when ripe and have a highly refined eating quality. Often in the breeding process, certain characteristics are prioritized at the expense of others in each market class. This contrast is particularly pronounced with oilseed pumpkins, where the fruit is essentially inedible because efforts have focused primarily on improving seed characteristics. These extremes in fruit phenotypes resulting from divergent breeding for fruit or seed consumption provide an excellent opportunity to study the genetic basis of fruit quality in winter squash.

Fruit quality is one of the most important breeding goals for winter squash. Although fruit quality involves the complex interplay of flavor, texture and appearance, there are three easily measured metabolites that have a major impact on quality. First, carotenoids are the source of winter squash fruit color and provide some of the greatest nutritional benefits of eating squash. The primary carotenoids in *C*. *pepo* are β-carotene and lutein.^1,2^ Second, starch content is important for winter squash fruit texture; ideally squash have a smooth, slightly dry consistency, which is correlated with higher starch and dry matter content.^3^ Two negative texture attributes, fibrousness and wateriness, are correlated with low starch and dry matter.^3^ Third, sugar content is correlated with perceived sweetness of winter squash, with sucrose as the most important predictor.^4^ Sweetness is thought to be important for consumer acceptability and to influence perception of overall squash flavor, as squash flavor was positively correlated with perceived sweetness in taste panels.^5^ Percent soluble solids, an efficient trait to phenotype, is also correlated with sucrose levels and perceived sweetness.^6^ Therefore, by measuring carotenoids, starch and sugar, it is possible to capture many of the important aspects of fruit quality.

These three fruit quality metabolites are synthesized and accumulate in predictable patterns throughout fruit development. The first stage in fruit development is fruit expansion, where the new fruit quickly grows to reach its maximum size 15–24 days after pollination (DAP). At the same time as fruit expansion, starch accumulates in the growing fruit, reaching maximum dry matter around 30 DAP. At this time, sugars start to accumulate, and eventually, especially after harvest, starch is degraded and sugars continue to accumulate.^7,8^ Carotenoids also steadily accumulate after 30 DAP and in storage.^6,9^

Transcriptome sequencing can provide valuable insight into the gene expression underlying developmental changes during fruit development. It has been used to study fruit development and metabolite accumulation in tomato,^10^ pepper,^11,12^ grape,^13^ banana^14^ and more. Such studies have successfully captured the expression of metabolic genes involved in the biosynthesis of the metabolites of interest. This technique is especially useful in squash (2*n*=2*x*=40), because it does not require a completed genome assembly; there is currently no genome assembly available for squash, which is estimated to have a genome size of 500 Mb.^15^ Previous squash transcriptome studies have included analyses of summer squash (*C. pepo*) root, leaf and flower tissue,^16^ pumpkin (*C. pepo*) leaf and flower tissue,^17^ *C. moschata* leaf, stem and shoot tissue,^18^ and acorn squash (*C. pepo*) fruit and seed tissue.^19^ No study to date, however, has used transcriptome sequencing to look at differential gene expression throughout winter squash fruit development.

In this study, we describe comparative transcriptome sequencing throughout fruit development of two squash bred for different end uses at the mature fruit stage. The first cultivar we sequenced was bred for consumption of the fruit mesocarp: ‘Sweet REBA’. ‘Sweet REBA’ is an pureline acorn squash cultivar with a reference fruit and seed transcriptome that we previously generated.^19^ We also sequenced ‘Lady Godiva’, an oilseed pumpkin that was bred for the consumption of its hull-less seeds and whose mesocarp is not intended for consumption and is discarded after seed extraction. Sequencing their fruit transcriptome at five time points allowed us to examine how metabolic processes act throughout mesocarp fruit development to create squash with contrasting fruit quality phenotypes.

## Materials and methods

### Plant material

‘Sweet REBA’ acorn squash and an inbred line derived from the OP oilseed pumpkin ‘Lady Godiva’ were chosen for this transcriptomic analysis of winter squash fruit quality because they are both inbred lines that are grown commercially as cultivars, which makes them representative of squash in the marketplace. They are both within the same species, *C. pepo*, which allowed us to make use of the wider array of genetic resources available in this species. In addition, they were bred for contrasting end uses, reflected in our previous observation of their large differences in fruit quality, which suggested they would provide a useful contrast of fruit quality traits throughout fruit development. Both lines were grown at the Homer C. Thompson Vegetable Research Farm (Freeville, NY, USA) in summer 2012 using standard horticultural practices. Flowers were manually self-pollinated and mesocarp and seed tissue were collected from fruits at 5, 10, 15, 20 and 40 DAP. Mesocarp samples were taken from a longitudinal slice of the fruit, excluding seed, placental and rind tissue. Approximately 40 ml of chopped tissue per sample was flash-frozen in liquid nitrogen, stored at −80 °C, and used for RNA sequencing and carotenoid analysis. Additional longitudinal slices of mesocarp tissue were used for soluble solids and dry matter analysis. Seed tissue was collected and used for RNA sequencing and transcriptome assembly, but was not used further in this study. Three biological replicates were collected for each genotype per time point, each consisting of two fruits from different plants.

### Fruit metabolite phenotyping

Fruit quality traits were phenotyped using mesocarp samples from the same fruits used for RNA sequencing. Carotenoids were measured with high-performance liquid chromatography using the protocol described in Van Eck *et al.*^20^ Percent soluble solids (degrees Brix) was determined by freezing samples, allowing them to thaw and analyzing their juice using a digital refractometer. Percent dry matter was calculated by drying mesocarp samples at 60 °C until they reached a constant weight and then dividing the final weight by the starting weight.

### RNA extraction and sequencing

Total RNA was extracted from mesocarp and seed samples using a Qiagen RNeasy Plant Mini Kit (Qiagen, Valencia, CA, USA). For each biological replicate, equal amounts of total RNA from two fruit were pooled. The Indiana University Genomics Facility prepared barcoded sequencing libraries using the Illumina TruSeq Sample Prep Kit (Illumina, San Diego, CA, USA) and samples were sequenced on three lanes of an Illumina HiSeq 2000.

### Transcriptome assembly

Reads from ‘Sweet REBA’ from a previous analysis^19^ were combined with the reads sequenced in this study for transcriptome assembly. Reads were cleaned with Fastq-mcf^21^ with a minimum read length of 50 bp and minimum base quality score of 30. Read quality was assessed with FastQC^22^ before and after cleaning. Reads from the same genotype were assembled together with Trinity v2.0.6^23^ to result in two assemblies. Corset v1.03^24^ was used to assign genes into clusters and the longest transcript was selected from each cluster to use as the representative transcript. Contigs from ‘Lady Godiva’ were queried against the ‘Sweet REBA’ assembly with BLAST^25^ and any contigs not found or with <90% sequence identity were added to the combined assembly. This representative transcriptome was cleaned of contaminating sequences with SeqClean,^26^ using plant pathogen sequences as a screening database.^27^

### Transcription annotation and analysis

The transcriptome was screened against the SWISS-PROT, TrEMBL^28^ and TAIR10^29^ protein databases using BLASTx with a cutoff of 1e^−20^ to assign putative functions to transcripts. Predicted peptides were analyzed using InterProScan^30^ to identify functional protein domains and assign Gene Ontology (GO) terms.

Gene expression in fruit mesocarp samples from ‘Sweet REBA’ and ‘Lady Godiva’ was analyzed by mapping reads to the representative transcriptome using bowtie2,^31^ then using Cuffdiff^32^ to calculate FPKM (fragments per kilobase of exon per million reads mapped) values and identify significantly differentially expressed genes. Expression differences were considered significant for *P *values less than the false discovery rate as determined by the Benjamini–Hochberg correction for multiple tests.^32^ Carotenoid, sugar and starch metabolic gene homologs were identified based on their annotations and using BLAST.^25^ FPKM values were log transformed using log2+1 and plotted using the R heatmap.2 function in the gplots package.^33^

The data sets were then filtered using the following criteria: (1) a significant *Q* value, (2) expression value greater than 1 FPKM in at least one stage of development and (3) a log2 fold change greater than 2. A Venn diagram was created to determine overlapping sets of filtered, differentially expressed genes between ‘Sweet REBA’ and ‘Lady Godiva’ at the five stages of development.^34^ To see which filtered genes showed similar expression over time in each accession, K-means clustering was performed using the Gap statistic^35^ to determine the number of clusters. The R packages cluster^36^ and factoextra^37^ were used for clustering. Overrepresented GO terms were investigated for each cluster using a hypergeometric test and the Benjamini and Hochberg False Discovery Rate as implemented in the BiNGO^38^ plugin for Cytoscape.^39^

## Results

### Fruit metabolite phenotypes

**Figure 1:**
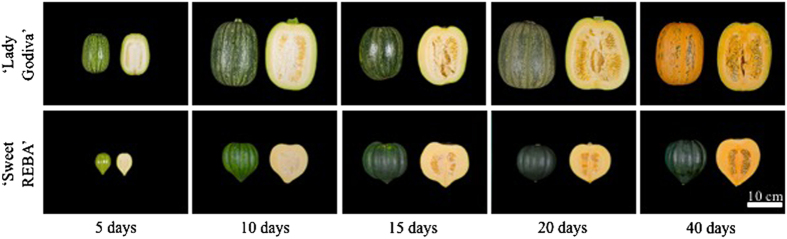
‘Lady Godiva’ oilseed pumpkin and ‘Sweet REBA’ acorn squash at five developmental time points. Self-pollinated fruit were collected at 5, 10, 15, 20 and 40 days after pollination. Photos are of the interior and exterior of representative fruit at each time point.

**Figure 2:**
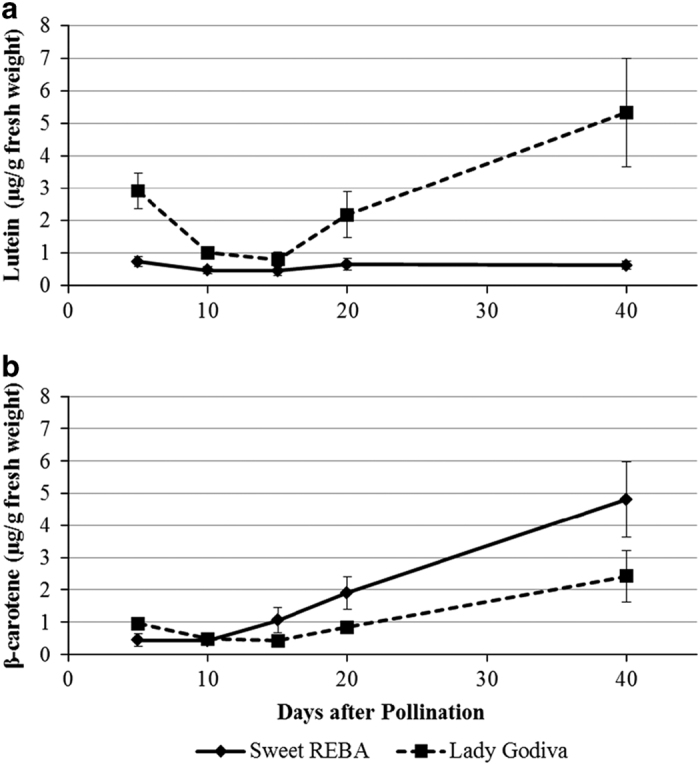
Carotenoid concentrations of ‘Sweet REBA’ and ‘Lady Godiva’ squash throughout fruit development. Self-pollinated fruit were collected at 5, 10, 15, 20 and 40 days after pollination. All measurements were performed on fruit mesocarp and are averages of six fruit. Error bars indicate the s.d. Lutein (**a**) and β-carotene (**b**), measured using high-performance liquid chromatography.

**Figure 3:**
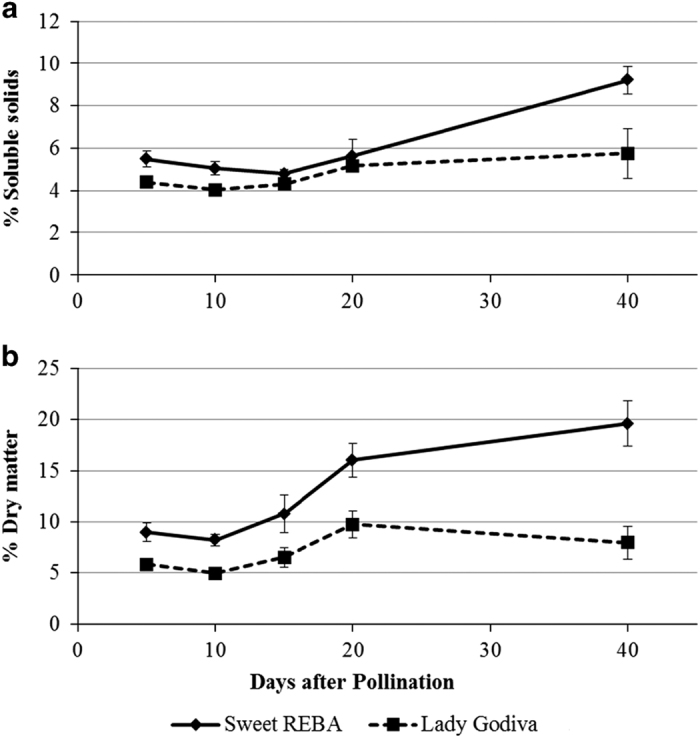
Percent soluble solids and percent dry matter of ‘Sweet REBA’ and ‘Lady Godiva’ squash throughout fruit development. Self-pollinated fruit were collected at 5, 10, 15, 20 and 40 days after pollination. All measurements were performed on fruit mesocarp and are averages of six fruit. Error bars indicate the s.d. Percent soluble solids (**a**), measured using a refractometer. Percent dry matter (**b**), measured through comparison of wet and dry weights.

Self-pollinated fruits were collected from the inbred acorn squash cultivar ‘Sweet REBA’ and an inbred line derived from the OP oilseed pumpkin ‘Lady Godiva’ at 5, 10, 15, 20 and 40 DAP (Figure 1) and mesocarp tissue was analyzed to determine the concentration of important fruit quality metabolites at each time point. The two primary carotenoids detected in the mesocarp were lutein and β-carotene. The lutein content of ‘Lady Godiva’ was much higher than ‘Sweet REBA’, with more than 5 μg g^−1^ fresh weight by 40 DAP (Figure 2a). Conversely, ‘Sweet REBA’ accumulated approximately twice as much β-carotene as ‘Lady Godiva’ (Figure 2b). Overall, ‘Lady Godiva’ accumulated a greater amount of carotenoids. In contrast, ‘Sweet REBA’ had higher sugar, measured as percent soluble solids, at all five developmental time points (Figure 3a). Both cultivars had soluble solids that ranged between 4 and 6% for the first 20 days of fruit development, but by 40 DAP, ‘Sweet REBA’ had more than 9% soluble solids while ‘Lady Godiva’ stayed near 6%. A similar pattern was seen in starch levels, measured as percent dry matter (Figure 3b). ‘Sweet REBA’ had a higher dry matter at all time points, with an especially large contrast with ‘Lady Godiva’ by 40 DAP.

### Transcriptome sequencing and assembly

RNA was extracted from the mesocarp samples used for phenotypic analysis, as well as from the seeds of the same fruit. Three biological replicates each were sequenced for both mesocarp and seed tissue of each genotype at each of the five developmental time points. Each biological replicate was made up of pooled RNA from two fruits. Barcoded RNA sequencing libraries were sequenced on three lanes of an Illumina HiSeq 2000 (reads available in the NCBI Sequence Read Archive repository, #SRP082555). The resulting reads were combined with ‘Sweet REBA’ reads from a previous analysis^19^ and Trinity^23^ was used to make two genotype-specific assemblies (Table 1), which were then clustered using Corset^24^ and the longest transcript in each cluster was chosen to use as the representative transcript. The two sets of unigenes were then compared with each other using BLAST^25^ and the full ‘Sweet REBA’ assembly was combined with the ‘Lady Godiva’ unigenes not found in the ‘Sweet REBA’ assembly to make the final transcriptome, resulting in a total of 63 175 unigenes in the transcriptome with an average length of 1454 base pairs (This Transcriptome Shotgun Assembly project has been deposited at DDBJ/EMBL/GenBank under the accession GEYH00000000. The version described in this paper is the first version, GEYH01000000; annotation and other data available in Supplementary Table S1).

### Metabolite pathway analysis

Proposed pathways for carotenoid, starch and sugar biosynthesis during winter squash fruit development were assembled from the literature and one or more homologs for all of the structural genes involved were identified using annotation data and BLAST. For instances where multiple homologs were identified, the homolog that was the best fit for our proposed biosynthetic pathway and most consistent with the observed phenotypic differences between the two cultivars was selected. Expression of these fruit quality biosynthetic genes was calculated from the fruit mesocarp transcriptome data across five time points in fruit development.

**Figure 4:**
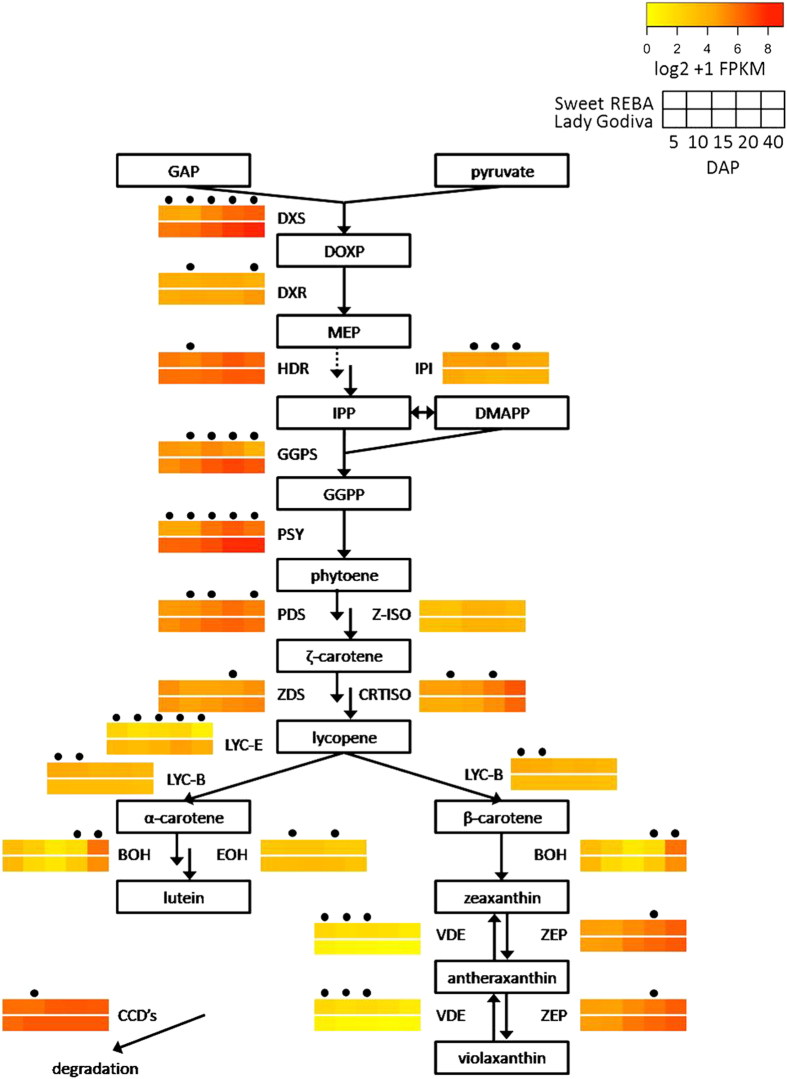
Carotenoid metabolism and associated gene expression. Proposed pathway of carotenoid metabolism in developing winter squash fruit, as derived from the literature.^40–42^ For each step, gene expression throughout fruit development is displayed for the best candidate homolog in our fruit and seed transcriptome. Gene expression is displayed as heat maps depicting the log2+1 transformation of FPKM (fragments per kilobase of exon per million reads mapped) values for ‘Sweet REBA’ (top heat map) and ‘Lady Godiva’ (bottom heat map) at 5, 10, 15, 20 and 40 DAP (days after pollination). Time points with a dot above them are significantly differentially expressed (*P*<FDR after Benjamini-Hochberg correction) between the two genotypes. Pathway genes, abbreviations and best candidates are as follows: DOXP synthase, DXS, CP437084; DOXP reductoisomerase, DXR, CP420725; 4-hydroxy-3-methylbut-2-enyl diphosphate reductase, HDR, CP430787; IPP isomerase, IPI, CP408939; GGPP synthase, GGPS, CP410963; phytoene synthase, PSY, CP426243; phytoene desaturase, PDS, CP443724; z-carotene isomerase, Z-ISO, CP413554; z-carotene desaturase, ZDS, CP432980; carotenoid isomerase, CRTISO, CP422707; lycopene β-cyclase, LYC-B, CP420557; lycopene ε-cyclase, LYC-E, CP434467; ε-hydroxylase, EOH, CP412995; β-carotene hydroxylase, BOH, CP444787; zeaxanthin epoxidase, ZEP, CP446828; violaxanthin de-epoxidase, VDE, CP411821; carotenoid cleavage dioxygenase, CCD, CP427752. Metabolite abbreviations and names are as follows: DMAPP, dimethylallyl diphosphate; DOXP, 1-deoxyxylulose 5-phosphate; GAP, glyceraldehyde-3-phosphate; GGPP, geranylgeranyl diphosphate; IPP, isopentenyl diphosphate; MEP, 2-C-methyl-D-erythritol 4-phosphate.

Homologs were found for all genes in the well-characterized carotenoid pathway.^40–42^ Most of the genes were either more highly expressed in ‘Lady Godiva’ or had approximately equal expression levels in the two cultivars (Figure 4). Some exceptions were IPP isomerase (IPI), carotenoid isomerase (CRTISO), lycopene β-cyclase (LYC-B), β-carotene hydroxylase (BOH), and violaxanthin de-epoxidase (VDE), which had a general pattern of higher expression in ‘Sweet REBA’ and were significantly higher expressed in at least two time points. DOXP synthase (DXS), GGPP synthase (GGPS), phytoene synthase (PSY) and carotenoid cleavage dioxygenase (CCD) were more highly expressed than the other genes in the pathway, with FPKM values exceeding 100 for ‘Lady Godiva’, while the other genes averaged an overall FPKM of approximately 25. In addition, four genes in the pathway were significantly differentially expressed between ‘Sweet REBA’ and ‘Lady Godiva’ at most time points through fruit development: DXS, GGPS, PSY and lycopene ε-cyclase (LYC-E).

**Figure 5:**
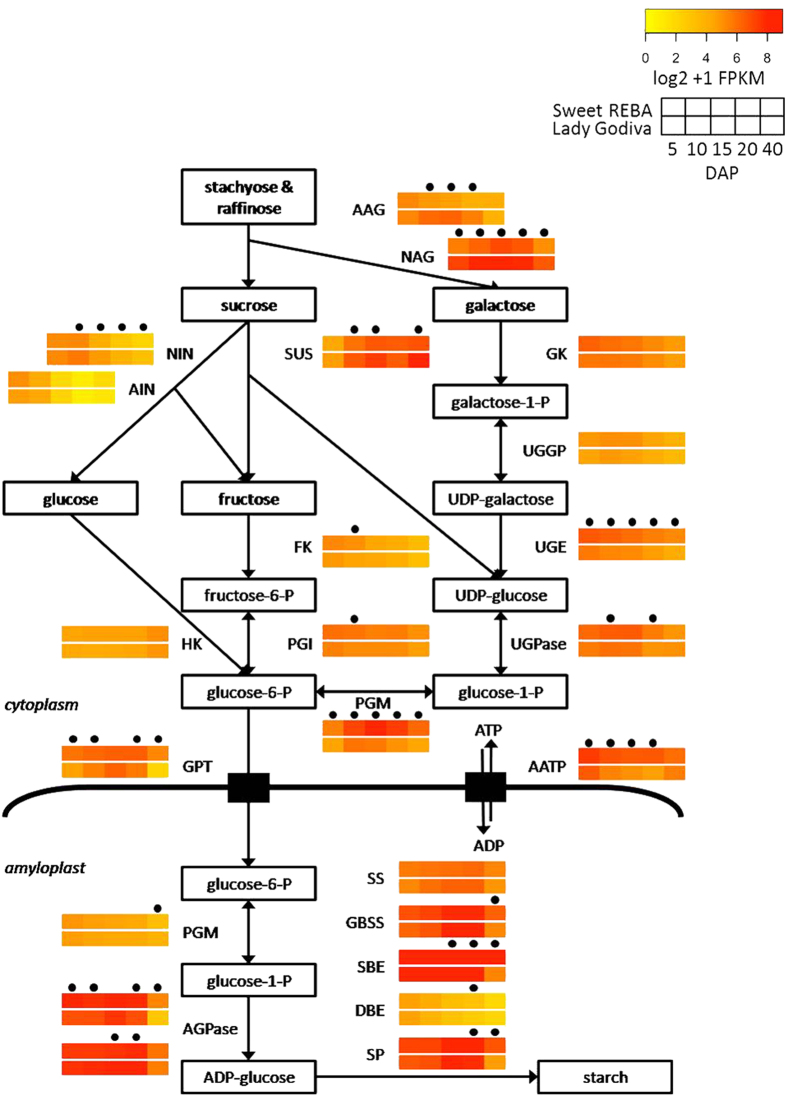
Starch biosynthesis and associated gene expression. Proposed pathway of starch biosynthesis in developing winter squash fruit, as derived from the literature.^7,43–46^ For each step, gene expression throughout fruit development is displayed for the best candidate homolog in our fruit and seed transcriptome. Gene expression is displayed as heat maps depicting the log2+1 transformation of FPKM (fragments per kilobase of exon per million reads mapped) values for ‘Sweet REBA’ (top heat map) and ‘Lady Godiva’ (bottom heat map) at 5, 10, 15, 20 and 40 DAP (days after pollination). Time points with a dot above them are significantly differentially expressed (*P*<FDR after Benjamini-Hochberg correction) between the two genotypes. Metabolites in bold are those imported into the fruit.^47^ Pathway genes, abbreviations, and best candidates are as follows: acid α-galactosidase, AAG, CP408004; alkaline α-galactosidase, NAG, CP446811; galactokinase, GK, CP441095; UDP-glucose/galactose pyrophosphorylase, UGGP, CP418531; UDP-glucose epimerase, UGE, CP432008; acid invertase, AIN, CP415669; neutral invertase, NIN, CP434838; sucrose synthase, SUS, CP432631; hexokinase, HK, CP425900; fructokinase, FK, CP412239; phosphoglucose isomerase, PGI, CP414400; UDP-glucose pyrophosphorylase, UGPase, CP434649; phosphoglucomutase-cytoplasmic, PGM, CP442762; glucose phosphate transporter, GPT, CP427241; amyloplastidial ATP/ADP translocator, AATP, CP429905; phosphoglucomutase-plastidic, PGM, CP441867; ADP-glucose pyrophosphorylase, AGPase, CP440578 (large subunit, top heat map), CP426204 (small subunit, bottom heatmap); soluble starch synthase, SS, CP446376; granule-bound starch synthase, GBSS, CP444286; starch-branching enzyme, SBE, CP446947; debranching enzyme, DBE, CP443615; starch phosphorylase, SP, CP425595.

A putative starch biosynthesis pathway for winter squash fruit was assembled from the literature.^7,43–46^ Imported sugars stachyose, raffinose, sucrose, galactose, glucose and fructose^47^ are converted into glucose-6-P, which is imported into the amyloplast and used to synthesize starch. Homologs were found for all of the genes in our proposed pathway and in ‘Sweet REBA’, most of the genes were expressed at a level equal to or higher than ‘Lady Godiva’ (Figure 5). Some exceptions were acid α-galactosidase (AAG), alkaline α-galactosidase (NAG), neutral invertase (NIN), sucrose synthase (SUS) and the small subunit of ADP-glucose pyrophosphorylase (AGPase), which were significantly more highly expressed in ‘Lady Godiva’ in two or more time points. Two genes with much higher expression than the others in both cultivars were starch-branching enzyme (SBE) and starch phosphorylase (SP). Starch-branching enzyme in particular had an average FPKM value of more than 1 200, which was more than 9-fold higher than the overall average of all of the starch biosynthesis genes. Several genes were significantly differentially expressed between the two cultivars at four or more time points: NAG, UDP-glucose epimerase (UGE), NIN, phosphoglucomutase (PGM), glucose phosphate transporter (GPT), the amyloplastidial ATP/ADP translocator (AATP) and the large subunit of AGPase.

**Figure 6:**
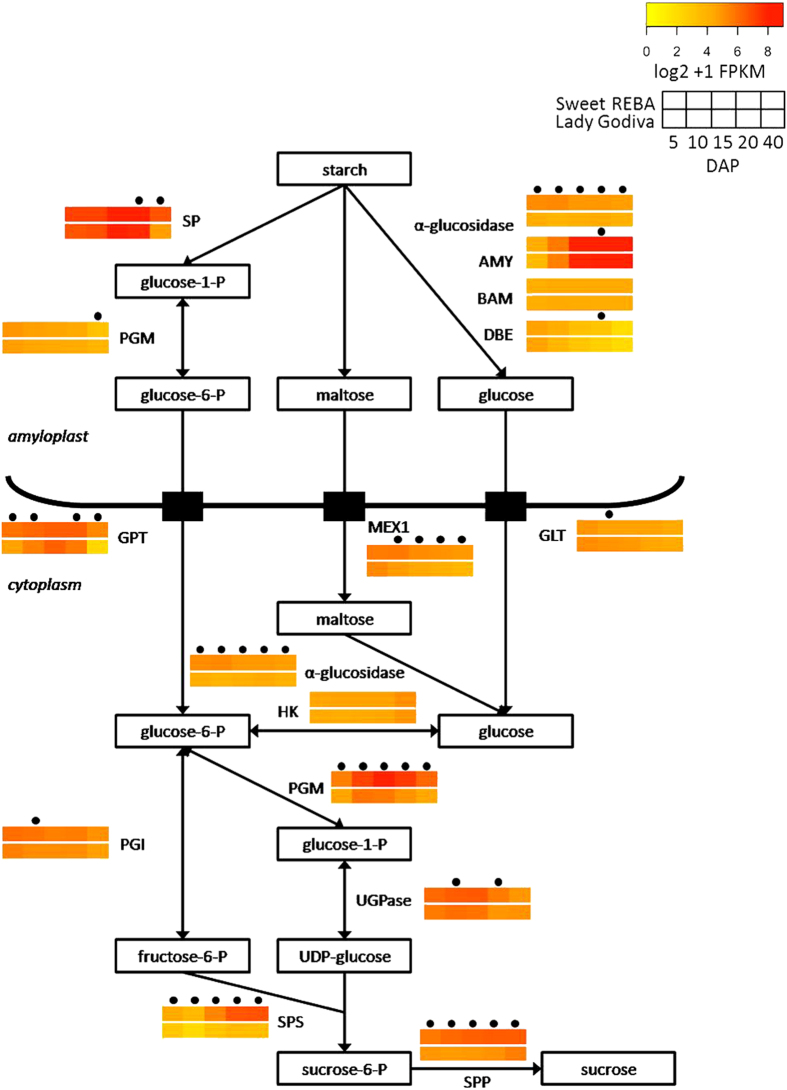
Sucrose biosynthesis and associated gene expression. Proposed pathway of starch breakdown and sucrose biosynthesis in developing winter squash fruit, as derived from the literature.^44,48–51^ For each step, gene expression throughout fruit development is displayed for the best candidate homolog in our fruit and seed transcriptome. Gene expression is displayed as heat maps depicting the log2+1 transformation of FPKM (fragments per kilobase of exon per million reads mapped) values for ‘Sweet REBA’ (top heat map) and ‘Lady Godiva’ (bottom heat map) at 5, 10, 15, 20 and 40 DAP (days after pollination). Time points with a dot above them are significantly differentially expressed (*P*<FDR after Benjamini-Hochberg correction) between the two genotypes. Pathway genes, abbreviations, and best candidates are as follows: α-amylase, AMY, CP435378; β-amylase, BAM, CP429571; debranching enzyme, DBE, CP443615; α-glucosidase, α-glucosidase, CP413233; starch phosphorylase, SP, CP425595; phosphoglucomutase-plastidic, PGM, CP441867; glucose phosphate transporter, GPT, CP427241; MEX1, MEX1, CP427138; plastidic glucose transporter, GLT, CP419962; hexokinase, HK, CP425900; phosphoglucomutase-cytoplasmic, PGM, CP442762; UDP-glucose pyrophosphorylase, UGPase, CP434649; phosphoglucose isomerase, PG, CP414400; sucrose-phosphate synthase, SPS, CP447452; sucrose-phosphate phosphatase, SPP, CP417885.

After squash reach a maximum dry weight, starch is hydrolyzed over the remainder of fruit development and storage and the resulting sugars are used to synthesize sucrose. We assembled a putative pathway for this process from the literature^44,48–51^ and found homologs in our fruit transcriptome for all of the genes involved. As with the proposed starch synthesis pathway, most of the genes in the sucrose synthesis pathway were either more highly expressed in ‘Sweet REBA’ or were equally expressed in both cultivars (Figure 6). SP, as in the starch biosynthesis pathway, was very highly expressed in both cultivars. Six of the genes involved in starch breakdown and sucrose synthesis were significantly differentially expressed at four or five of the five developmental time points: α-glucosidase, GPT, MEX1, PGM, sucrose-phosphate synthase (SPS) and sucrose-phosphate phosphatase (SPP).

### Differential gene expression analysis

**Figure 7:**
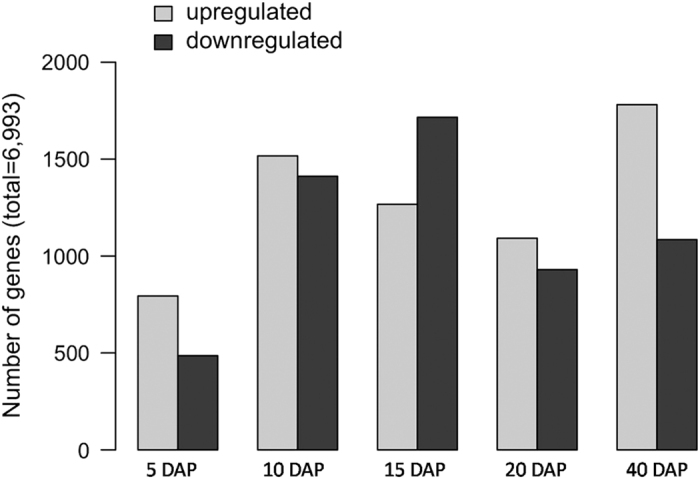
Differential gene expression in ‘Sweet REBA’ and ‘Lady Godiva’ squash fruit at five developmental time points. ‘Sweet REBA’ and ‘Lady Godiva’ unigene expression levels were compared at five developmental time points in three replicates. The number of significantly differentially expressed unigenes that were upregulated in ‘Lady Godiva’ as compared with ‘Sweet REBA’ (‘upregulated’) or downregulated in ‘Lady Godiva’ as compared with ‘Sweet REBA’ (‘downregulated’) at each time point are displayed.

Reads were mapped to the assembled transcriptome using bowtie2 (Supplementary Table S2) and then Cuffdiff was used to identify unigenes differentially expressed between genotypes at each developmental time point. The unigenes were filtered to retain only unigenes with an FPKM value of at least 1 for at least one time point (for the time point comparisons), a significant *Q* value, and a log2 fold change greater than 2. This resulted in a total of 6 993 unigenes differentially expressed in at least one time point comparison (Figure 7; Supplementary Figure S1). Less unigenes were differentially expressed at 5 DAP, and more were differentially expressed at 10, 15 and 40 DAP. The 40 DAP time point had the greatest difference between genotypes, with many more unigenes upregulated in ‘Lady Godiva’ as compared with ‘Sweet REBA’. BiNGO was then used to identify GO terms overrepresented in the differentially expressed genes at each time point (Supplementary Table S3). The 40 DAP time point also had the greatest number of overrepresented GO terms. ‘Sweet REBA’ upregulated unigenes were enriched for carbohydrate metabolism-related GO terms such as ‘glucose metabolic process’, ‘glucan biosynthetic process’, and ‘sucrose metabolic process’. ‘Lady Godiva’ upregulated unigenes, in contrast, were enriched for ‘pigment metabolic process’, among many others.

### Clustering analysis

**Figure 8:**
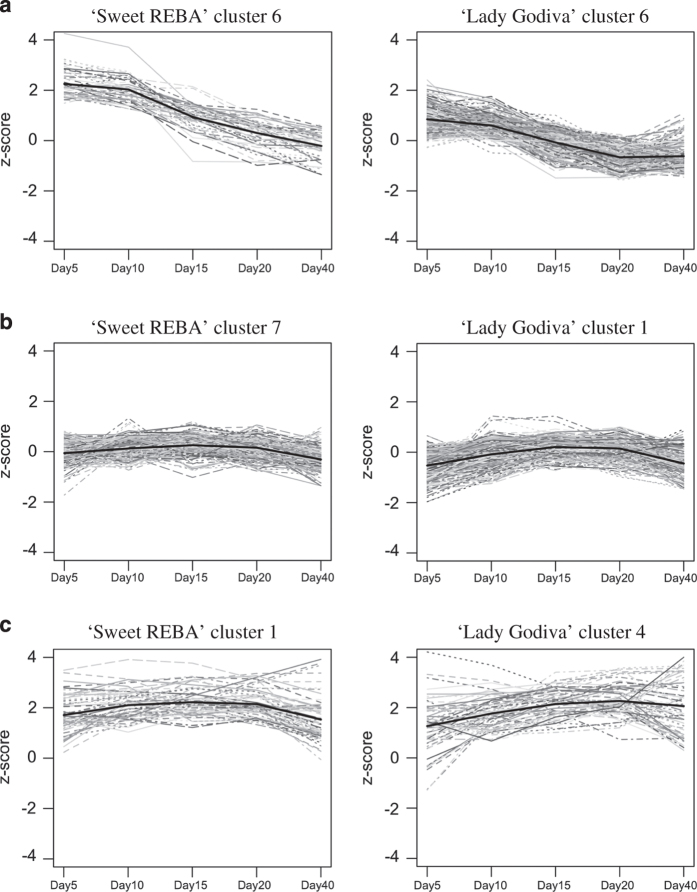
Select clustered unigene expression profiles across ‘Sweet REBA’ and ‘Lady Godiva’ squash fruit development. ‘Sweet REBA’ and ‘Lady Godiva’ unigenes that were differentially expressed across fruit development were clustered via K-means clustering using the Gap statistic. Select clusters of interest are displayed which were enriched for unigenes related to (**a**) negative regulation of metabolic processes, (**b**) carbohydrate metabolism and cell wall biosynthesis and (**c**) carbohydrate metabolism.

Cuffdiff was also used to identify unigenes differentially expressed across the five time points within each genotype, using the same filtering described above. These unigenes were then clustered by expression profile across fruit development using K-means clustering using the Gap statistic to determine the number of clusters. This resulted in 10 clusters for ‘Sweet REBA’ and seven clusters for ‘Lady Godiva’ (Supplementary Figures S2 and S3). There were corresponding expression patterns for clusters across the two genotypes, such as ‘Sweet REBA’ cluster 5 versus ‘Lady Godiva’ cluster 7 and ‘Sweet REBA’ cluster 8 versus ‘Lady Godiva’ cluster 2. BiNGO was then used to identify GO terms overrepresented in each cluster (Supplementary Table S4). The types of GO terms varied by cluster, but clusters with similar expression patterns tended to be overrepresented for similar types of GO terms. For example, cluster 6 in both genotypes had an expression pattern that decreased throughout fruit development and were both enriched for GO terms related to negative regulation of metabolic processes (Figure 8a). ‘Sweet REBA’ cluster 7 and ‘Lady Godiva’ cluster 1 were both enriched for carbohydrate metabolism and cell wall biosynthesis GO terms and had expression patterns that remained fairly constant at average levels throughout fruit development (Figure 8b). Finally, ‘Sweet REBA’ cluster 1 and ‘Lady Godiva’ cluster 4 had expression patterns that were above average across fruit development and were enriched for carbohydrate metabolism GO terms (Figure 8c).

## Discussion

### *C. pepo* fruit and seed transcriptome assembly

In this study, we successfully sequenced and assembled new transcriptome data from *C. pepo* fruit and seeds throughout development. Our updated fruit and seed transcriptome now contains data from the oilseed pumpkin ‘Lady Godiva’ and has a slightly longer average unigene length, 1454 bp as compared with 1315 bp in the previous fruit and seed transcriptome.^19^ This could indicate a greater number of full-length unigenes in this transcriptome, potentially made possible by the greater amount of sequence data used in this assembly. This updated transcriptome has a greater number of unigenes than two of the published *C. pepo* transcriptomes,^16,19^ which could be due to an inability to collapse the different alleles sequenced from the two genotypes for some genes. When the in-progress *C. pepo* genome sequencing effort is completed, use of the genome will enable us to reduce the number of unigenes to accurately reflect the number of genetic loci, removing redundancy. Based on our identification of homologs of all the metabolic genes of interest, the transcriptome successfully captured gene expression throughout winter squash fruit development.

### Fruit metabolite phenotypes

Our phenotypic analysis revealed the expected differences in the fruit quality metabolites of ‘Sweet REBA’ and ‘Lady Godiva’, which were bred for different purposes. The two primary carotenoids detected were lutein and β-carotene, as found in previous *C. pepo* carotenoid analyses.^1,2,52,53^ The detected increase in carotenoid levels over fruit development was also found in studies of carotenoid accumulation in *C. pepo*^53^ and *C. moschata* and *C. maxima.*^54^ The final lutein and β-carotene concentration in our two cultivars was within the range reported for *C. pepo* in the literature: 0–23 μg g^−1^ β-carotene and 0–10 μg g^−1^ lutein.^1,2,52^ We were surprised to find that ‘Lady Godiva’ accumulated more total carotenoids than ‘Sweet REBA’ since the flesh of this fruit was not bred for consumption, but it is still well within the range of normal variation for *C. pepo*. The higher carotenoid levels of ‘Lady Godiva’ could be a pleiotropic effect of selecting for dark green seed and seed oil color, traits that are prized in oilseed pumpkins.

The two carbohydrate metabolite analyses we conducted revealed additional differences in fruit quality traits between the two cultivars. ‘Sweet REBA,’ which was bred for consumption of fruit mesocarp, had a higher percent dry matter and percent soluble solids than ‘Lady Godiva’, which was selected for seed traits. Dry matter generally increased over fruit development, with a slight dip at 10 DAP, which is during a period of rapid fruit expansion. This increase in dry matter and starch content over fruit development is consistent with previous studies of starch accumulation in winter squash,^6,7,55,56^ and the higher dry matter in ‘Sweet REBA’ underlies its observed smoother texture. The percent soluble solids measured across fruit development is also in line with previous studies, in which soluble solids generally stayed at a low level and then started increasing around 40 DAP.^6,7,55^

### Comparative gene expression in the carotenoid pathway

Carotenoid structural gene expression levels corresponded with carotenoid accumulation observed across fruit development. This pattern is shared in other cucurbits, as reported in summer squash^53^ and watermelon.^57^ ‘Lady Godiva’ had both higher total carotenoid concentration and higher expression of carotenoid biosynthesis genes, with seven out of the sixteen genes significantly differentially expressed at the majority of time points sampled. This correlation between carotenoid gene expression and levels of carotenoid accumulation has also been observed when comparing both between cultivars of summer squash and between different fruit tissues within a cultivar,^53^ between *C. moschata* and *C. maxima,*^54^ and between watermelon cultivars with different fruit flesh colors.^58,59^ Taken together, these patterns suggest that transcriptional regulation is an important determinant of carotenoid accumulation in *C. pepo* winter squash, as it is in other squash market classes and other species.^53,58,59^

The differential carotenoid gene expression between ‘Lady Godiva’ and ‘Sweet REBA’ was especially pronounced for the genes known in other species to be important determinants of carotenoid synthesis and degradation. DOXP synthase (DXS), which is the first enzyme in the DOXP pathway that produces the precursors for carotenoids, is predicted to be a regulatory step for carotenoid synthesis in tomatoes.^60^ DOXP synthase had up to a threefold higher expression in ‘Lady Godiva’, especially later in fruit development when carotenoids were accumulating to higher concentrations, and was significantly different from ‘Sweet REBA’ at all five time points. Significant differential expression was also seen for GGPP synthase (GGPS). Another important step in carotenoid synthesis is PSY, which is a rate-limiting step in the carotenoid biosynthetic pathway in marigolds, canola, and tomato.^41^ Phytoene synthase was significantly more highly expressed in ‘Lady Godiva’, with 4-fold higher expression at 40 DAP, similar to what was observed in summer squash, where phytoene synthase was much more highly expressed in an orange flesh cultivar as compared with a white flesh cultivar^53^ and phytoene synthase expression was positively correlated with carotenoid content in some tissues and cultivars,^61^ and in *C. maxima*, which had a higher carotenoid concentration and also higher phytoene synthase expression than *C. moschata.*^54^ Phytoene synthase has also been suggested to be a central enzyme in controlling carotenoid concentration in watermelon.^62^ Finally, carotenoid breakdown is also an important determinant of carotenoid levels in fruit tissue. CCDs were not generally differentially expressed in the two cultivars, and thus did not likely influence their differential carotenoid concentrations. This is in contrast to some cultivars of summer squash, where levels of carotenoids in the fruit were inversely correlated with CCD expression.^63^

From lycopene, either both lycopene ε-cyclase (LYC-E) and lycopene β-cyclase (LYC-B) can act to make α-carotene, which is then converted to lutein, or solely lycopene β-cyclase can act to make β-carotene. In maize, natural variation in lycopene ε-cyclase influences the division of the carotenoid flux through the two branches of the pathway.^64^ In our study, the higher expression of lycopene β-cyclase in ‘Sweet REBA’ (significantly differentially expressed at two time points) is consistent with its higher accumulation of β-carotene and the higher expression of lycopene ε-cyclase in ‘Lady Godiva’ (significantly differentially expressed at all five time points) is consistent with its higher accumulation of lutein.

We observed a relatively high level of expression of β-carotene hydroxylase (BOH; at 40 DAP) and zeaxanthin epoxidase (ZEP), even though zeaxanthin and violaxanthin did not accumulate to appreciable levels in our squash. This is likely explained by the high levels of CCDs in both cultivars, which degrade carotenoids and could have kept the levels of zeaxanthin and violaxanthin low by degrading them quickly. This phenomenon was also described previously in watermelon.^57^

### Comparative gene expression in the starch synthesis pathway

In early fruit development, sugars must be transported to the fruit to provide the precursors for metabolite biosynthesis as well as other developmental processes. The two primary transport sugars in squash are stachyose and raffinose. Sucrose, galactose, glucose, and fructose are also transported.^47^ Alkaline and acid α-galactosidase (NAG, AAG) both act to break down stachyose and raffinose, and they had high expression levels early in fruit development in our study. Similar patterns of enzyme activity were observed for alkaline and acid α-galactosidase in developing buttercup squash,^7^ including the higher expression of alkaline α-galactosidase, which they hypothesized was the primary enzyme working to break down stachyose and raffinose.

Stachyose and raffinose are broken down into sucrose and galactose, which then must be converted to glucose-6-P in order to synthesize starch. Sucrose can be broken down by either acid and neutral invertases (AIN and NIN) or SUS. We observed higher levels of invertase expression in very early fruit development and higher levels of sucrose synthase expression for the remainder of development. In buttercup squash, invertase activity was also high for the beginning of fruit development and then decreased, which was suggested to provide increased osmotic potential early in development for the rapidly expanding fruit.^7^ In the same study, sucrose synthase activity started high and then declined, but then increased again during the ripening stage. This continued sucrose synthase activity later in fruit development was similar to what we observed. Galactose is broken down by the sequential activity of galactokinase (GK), UDP-glucose/galactose pyrophosphorylase (UGGP), and UDP-glucose epimerase (UGE), which were all expressed at a moderate to high level, especially in the early stages of fruit development. UDP-glucose pyrophosphorylase (UGPase) and PGM were also expressed at a high level, which is in accord with their role in synthesizing glucose-6-P, which is then imported into the amyloplast as the substrate for starch synthesis.

Overall, the expression of these sugar metabolism genes was generally higher in ‘Sweet REBA’, which likely acted to provide the substrate needed for ‘Sweet REBA’ to synthesize more starch than ‘Lady Godiva’. One exception was alkaline α-galactosidase, which was significantly more highly expressed in ‘Lady Godiva’ and was particularly highly expressed at 10 and 15 DAP. Because ‘Lady Godiva’ was not accumulating as much starch as ‘Sweet REBA’, it may have required higher levels of imported sugars for other developmental processes, such as cellulose synthesis. Sucrose synthase was significantly more highly expressed in ‘Lady Godiva’, and could have also been working to supply substrate for cellulose synthesis, as previously suggested in winter squash.^7^

To synthesize starch, glucose-6-P and ATP must be imported to the amyloplast by the glucose phosphate transporter and the amyloplastidial ATP/ADP translocator (AATP) respectively. AATP is a major rate-limiting enzyme for starch synthesis in potato^65,66^ and had a significantly higher expression in ‘Sweet REBA’, which could contribute to the greater starch accumulation in ‘Sweet REBA’. Other important regulatory steps in potato starch synthesis are PGM, which converts glucose-6-P imported into the amyloplast into glucose-1-P, and AGPase, which makes ADP-glucose, the first committed reaction in starch synthesis.^65,66^ Plastidic phosphoglucomutase had roughly similar expression in the two genotypes, but the large subunit of ADP-glucose pyrophosphorylase had a significantly higher expression in ‘Sweet REBA’. In another study of winter squash, ADP-glucose phosphorylase was thought to be important in determining the level of starch accumulation.^56^

Several different enzymes work together to synthesize starch from ADP-glucose. Granule-bound starch synthase (GBSS) makes amylose, which is the unbranched form of starch. Soluble starch synthase (SS), starch-branching enzyme (SBE), and debranching enzyme (DBE) work together to synthesize amylopectin, the branched form of starch. In *C. moschata* as compared with *C. maxima*, expression of granule-bound starch synthase was positively correlated with amylose content.^56^ Differential expression of granule-bound starch synthase and soluble starch synthase varied throughout fruit development, with higher expression in ‘Lady Godiva’ in the middle stages of fruit development, but higher expression in ‘Sweet REBA’ at the end of fruit development. Starch-branching enzyme was significantly more highly expressed in ‘Sweet REBA’ at the final three stages of development. These expression differences may correspond to differential ratios of amylose and amylopectin in these two cultivars.

### Comparative gene expression in the sucrose synthesis pathway

After winter squash fruit reach full size and maximum dry matter, they shift from accumulating starch to degrading starch and accumulating sucrose. During this shift, starch degradation genes increase in expression. We noted a large increase in α-amylase (AMY) expression but a relatively constant level of β-amylase (BAM) expression, similar to enzyme activity patterns observed in buttercup squash.^48^ A similar developmental transition occurs in kiwifruit, where α-amylase activity increases after peak starch accumulation and soluble solid levels start to rise.^67^ In banana, which also accumulates starch and converts it to sugar during ripening, α-amylase activity, and then α-glucosidase and β-amylase activity, coincided with the onset of ripening and starch degradation^68^ and β-amylase activity was correlated with a decrease in starch and an increase in total sugars.^69^ Alpha-amylase and α-glucosidase were significantly more highly expressed in ‘Sweet REBA’ at one or more time points, providing more substrate for sucrose synthesis.

Starch breakdown can be phosphorolytic or hydrolytic. Hydrolytic breakdown via α-amylase and β-amylase was thought to be the primary method in squash due to measurable α-amylase and β-amylase activity and the detection of maltose, a product of hydrolytic breakdown.^48^ We observed high α-amylase expression, but also high expression of SP, which is responsible for phosphorolytic starch breakdown. Starch phosphorylase, however, is also involved in starch synthesis, so because its expression was especially high during early-to-mid fruit development, perhaps it was primarily involved in starch synthesis, leaving hydrolytic breakdown as the predominant starch breakdown pathway in squash.

The products of starch breakdown are exported from the amyloplast and then converted into fructose-6-P and UDP-glucose, the two substrates for sucrose synthesis. The sugar transporters glucose phosphate transporter (GPT) and MEX1 were significantly more highly expressed in ‘Sweet REBA’, indicating that more starch breakdown products may have been leaving the amyloplast. In addition, most of the enzymes involved in the conversion to fructose-6-P and UDP-glucose were significantly upregulated in ‘Sweet REBA’ at one or more time points: α-glucosidase, PGM, UGPase and phosphoglucose isomerase (PGI).

The final steps of sucrose synthesis are the sequential action of sucrose-phosphate synthase (SPS) and sucrose-phosphate phosphatase (SPP) to yield sucrose. The same final steps in starch to-sugar conversion are seen in banana, where sucrose-phosphate synthase activity increases as starch is being degraded and sucrose starts to accumulate. Sucrose-phosphate synthase is a major enzyme that is associated with sugar accumulation.^70^ In melon, sucrose-phosphate synthase activity was correlated with sucrose accumulation between cultivars that accumulated different levels of sucrose.^71^ The same relationship was seen in watermelon, in which sucrose-phosphate synthase activity was higher in cultivars that accumulated more sucrose^72^ and was also differentially expressed across fruit development.^62^ Sucrose-phosphate synthase and sucrose-phosphate phosphatase were both significantly differentially expressed at all five developmental time points sampled, with higher expression in ‘Sweet REBA’.

Taken together, the identification of starch and sucrose metabolic genes and analysis of their differential gene expression demonstrates that ‘Sweet REBA’ accumulates more starch because of higher expression of sugar metabolism and starch synthesis genes. This higher level of stored starch, in combination with higher expression of starch breakdown and sucrose synthesis genes, then permits ‘Sweet REBA’ to accumulate more sucrose while maintaining a high level of starch.

### Differential gene expression analysis

Towards a global view of gene expression in developing winter squash fruits, we identified unigenes that were differentially expressed between ‘Sweet REBA’ and ‘Lady Godiva’ at each time point and determined which GO terms were enriched in these data sets. No GO terms directly related to fruit quality were enriched in any of the data sets except for 40 DAP. This is consistent with our knowledge about squash fruit development, because the first four time points are during the initial fruit expansion and accumulation of dry matter stages,^8^ which are less likely to vary between genotypes. The 40 DAP time point, in contrast, is after fruit expansion has completed and around the time fruits reach maximum dry matter.^8^ As the total dry matter content has a major effect on later fruit quality, this time point is when we would expect to see the gene expression differences responsible for fruit quality differences. In addition, the over-representation of differentially expressed fruit quality-related unigenes at 40 DAP is consistent with the phenotypic data in this study, which showed the highest contrast in fruit quality traits between the two genotypes at 40 DAP.

The specific fruit quality-related GO terms overrepresented at 40 DAP are also consistent with our observed phenotypes. The unigenes upregulated in ‘Sweet REBA’ are enriched for GO terms related to carbohydrate metabolism, including hexose, disaccharide, oligosaccharide, and polysaccharide metabolic processes. This pattern matches the higher expression in ‘Sweet REBA’ of the starch and sucrose synthesis genes described earlier, as well as ‘Sweet REBA’s greater accumulation of starch and sucrose as compared with ‘Lady Godiva’. The unigenes upregulated in ‘Lady Godiva’ are enriched for the ‘pigment metabolic process’ GO term, which is expected based on ‘Lady Godiva’s greater accumulation of carotenoid pigments and the higher expression of important carotenoid biosynthetic genes in ‘Lady Godiva’.

### Clustering analysis

After identifying unigenes differentially expressed across fruit development in either ‘Sweet REBA’ or ‘Lady Godiva’, we clustered the identified unigenes by expression pattern and looked at the GO term enrichment in each cluster. Overall, there was a pattern to the types of GO terms enriched in these unigenes. Some of the most commonly represented GO term categories were carbohydrate metabolism, cell wall development, and regulation of metabolic processes. This is in accord with the major processes occurring in fruit development: fruit growth and expansion and the accumulation of carbohydrate and other metabolites.

Generally, the GO term enrichment corresponded logically with the individual cluster expression patterns. ‘Sweet REBA’ cluster 6 and ‘Lady Godiva’ cluster 6 had expression patterns that decreased across fruit development and were enriched for GO terms related to negative regulation of metabolic processes. The decrease in negative regulation over time could explain the observed higher expression of some of the fruit quality biosynthetic genes towards the end of fruit development, as well as the higher accumulation of the fruit quality metabolites at the later time points. ‘Sweet REBA’ cluster 7 and ‘Lady Godiva’ cluster 1 were enriched for cell wall and carbohydrate metabolism-related GO terms. Although they maintained a fairly average expression across fruit development, they peaked slightly from 10–20 DAP, especially in ‘Lady Godiva’, which is the period of major fruit growth^8^ and thus active cell wall biosynthesis. ‘Sweet REBA’ cluster 1 and ‘Lady Godiva’ cluster 4 had a steady, higher than-average expression across fruit development and were enriched for carbohydrate metabolism-related GO terms. Some of the starch metabolism genes highlighted above were also members of one or both of these clusters. This above-average expression was consistent with carbohydrate’s role as the primary type of storage molecule in winter squash fruit.

## Conclusion

In this study, we sequenced the fruit transcriptome of an acorn squash, ‘Sweet REBA’, and an oilseed pumpkin, ‘Lady Godiva’, across fruit development to explore the key gene expression differences that underlie the contrasting metabolic processes that affect winter squash fruit quality. These two cultivars were developed for different purposes, fruit versus seed consumption, and accordingly had a resulting contrast in the important fruit quality traits of carotenoid content, percent dry matter, and percent soluble solids. Putative metabolic pathways for the synthesis of carotenoids, starch and sugar were assembled from the literature and expression of the structural genes of these pathways varied both across fruit development and between the two cultivars. The overall trend was increased expression of metabolic genes during times of metabolite accumulation and in the cultivar that accumulated higher metabolite levels. This reveals the transcriptional regulation of metabolic gene expression that may be an important determinant of fruit quality. It will be important to investigate the expression patterns of these genes in additional winter squash cultivars in future studies to determine the degree to which these gene expression profiles describe metabolisms that lead to improved fruit quality in winter squash in *C. pepo* and related species. Furthermore, this resource will be useful for molecular-informed breeding of oilseed pumpkins and squash with an edible mesocarp toward a goal of reducing food waste while maintaining oilseed quality and yield.

## Figures and Tables

**Table 1:** Summary of transcriptome assembly data

*Assembly data*	*'Sweet REBA' assembly*^a^	*'Lady Godiva' assembly*^a^	*Final transcriptome*
Number of paired-end reads	380 609 749	219 816 844	NA
Number of contigs	57 057	62 273	63 175
Average contig length (bp)	1540.8	1427.7	1454.3
% GC content	40.8	40.8	40.8
Longest contig (bp)	17 018	18 715	17 018
Total bases	87 912 129	88 905 997	91 872 276

Abbreviations: NA, not applicable.

a Contig data describes genotypic-specific assemblies after running Corset.

## Notes

LE Wyatt is now a squash breeder at Johnny’s Selected Seeds. The remaining authors declare no conflict of interest.
