# Supplementary figures and images for: Comparative analysis of Cucurbita pepo metabolism throughout fruit development in acorn squash and oilseed pumpkin
Source: Hortic Res. 2016 Sep 21;3:16045–. doi: 10.1038/hortres.2016.45 (PMC5030761; doi:10.1038/hortres.2016.45)

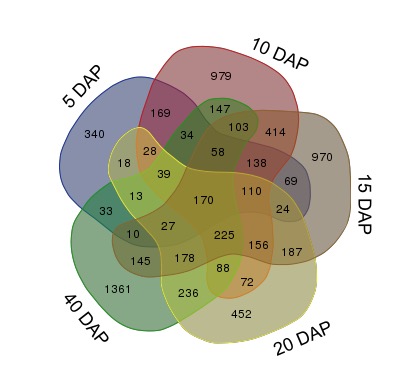

Supplement: Supplementary Figure S1 [file hortres201645-s1.jpg]

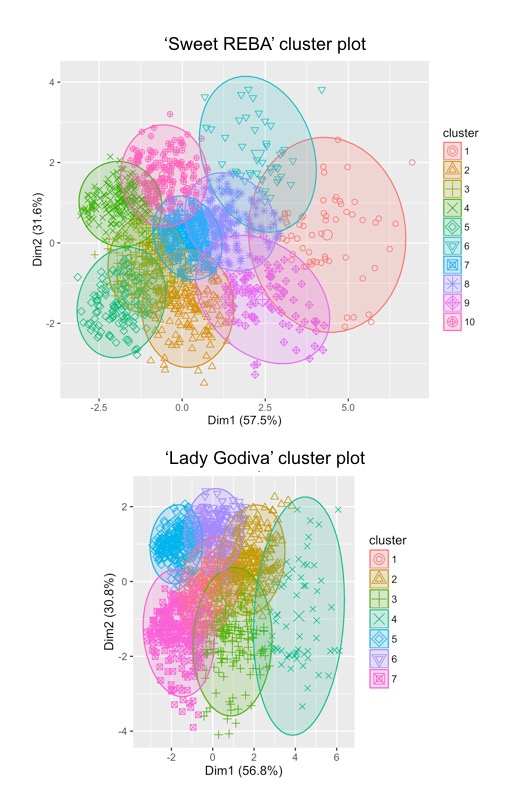

Supplement: Supplementary Figure S2 [file hortres201645-s2.jpg]

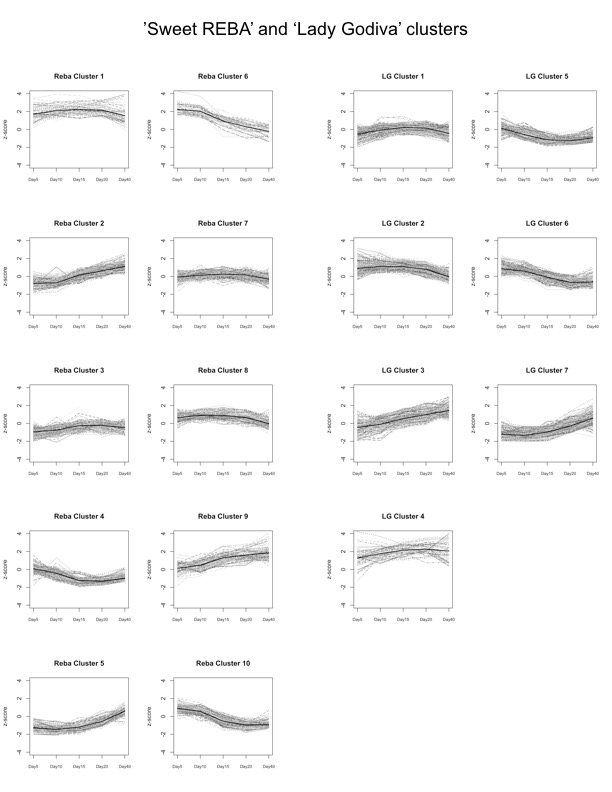

Supplement: Supplementary Figure S3 [file hortres201645-s3.jpg]
